# Outcomes Following Vascular and Endovascular Procedures Performed During the First COVID-19 Pandemic Wave

**DOI:** 10.1016/j.ejvsvf.2024.08.002

**Published:** 2024-09-19

**Authors:** Panagiota Birmpili, Ruth A. Benson, Brenig Gwilym, Sandip Nandhra, Nina Al-Saadi, Graeme K. Ambler, Robert Blair, David Bosanquet, Nikesh Dattani, Louise Hitchman, Katherine Hurndall, Matthew Machin, Sarah Onida, Athanasios Saratzis, Joseph Shalhoub, Lauren Shelmerdine, Aminder A. Singh, COVER Study Collaborative, Panagiota Birmpili, Ruth A. Benson, Brenig Gwilym, Sandip Nandhra, Nina Al-Saadi, Graeme K. Ambler, Robert Blair, David Bosanquet, Nikesh Dattani, Louise Hitchman, Katherine Hurndall, Matthew Machin, Sarah Onida, Athanasios Saratzis, Joseph Shalhoub, Lauren, Shelmerdine, Aminder Anthony Singh, Ruth A. Benson, Sandip Nandhra, Joseph Shalhoub, Graeme K. Ambler, Nikesh Dattani, David C. Bosanquet, Rachael O. Forsythe, Sarah Onida, George Dovell, Louise Hitchman, Ryan Preece, Athanasios Saratzis, Chris Imray, Sonia Kandola, Adam Johnson, Andrew Choong, Jun Jie Ng, Sarah Aitken, Jana-Lee Moss, Efthymios Beropoulis, Konstantinos Stavroulakis, Fabrico Santiago, Amr Abdelhaliem, Aseel Abuduruk, Thomas M. Aherne, Hazem Ahmed, Sarah J. Aitken, Tasleem Akhtar, Bekir B. Akkaya, Julien Al Shakarchi, Abdeljawad J. Algasi, Musaad AlHamzah, Ahmed A. Alhumiad, Bernard Allard, Meshal Almeshal, Faris Alomran, Reem N. AlRakaf, Mohamed Altabal, Nishath Altaf, Abdulmajeed H. Altoijry, Talal Altuwaijri, Nasser Alwehaibi, Sara J. Anderson Baker, Domenico Angiletta, Afroditi Antoniou, George A. Antoniou, Libnah L. Areias, James Ashcroft, Noel Atkinson, Doaa Attia, Lukas Attwell, Mohammed A. Azab, Omar Aziz, Ahmed Y. Azzam, Christos Bakoyiannis, Hashem Barakat, Khalid Bashar, Ruth Battersby, K.S. Benaragama, Ahmed T.S. BenGhatnsh, Nikolaos Bessias, Resya Bhakthavalsalan, Shagran Binkhamis, Roshan Bootun, Emily Boyle, Ion Buga, Martin Catterson, Jennifer L. Chambers, Karishma Chandarana, Alexandros Charalabopoulos, Gabriella Charlton, Stephen W.K. Cheng, Natasha Chinai, Asad J. Choudhry, Annie Clothier, Tina U. Cohnert, Chloe Coleman, Michael Costanza, Patrick A. Coughlin, James Coulston, James Cragg, Katy Darvall, Emma M. Davies, Huw Davies, Claire Dawkins, Joseph A. Dawson, Anastasia Dean, Bedanta S. Dhal, Andrew Duncan, Mark Edwards, Bridget Egan, Mehdi El Amrani, Ahmed Elhadi, Muhammed Elhadi, Mohamed S. Eljareh, Ramy Elkady, Mohamed Elkawafi, Fatimah S. Elkhafeefi, Maysoon Elkwahad, Ibrahim A. Ellojli, Kareem ElSanhoury, Hazem Elsantawy, Khaled Elsayed, Raed M. Ennab, Owain Fisher, Robert Fitridge, Ronald L.G. Flumignan, Amy L. Fowler, Richard F. Galloway, John Gan, Andrew Garnham, Sotirios Georgopoulos, Tamer M.H. Ghatwary Tantawy, Ravi R. Goel, Mingzheng A. Goh, Tabitha Grainger, Nalaka Gunawansa, Eric Hammond, Joseph Hanna, Simon C. Hardy, Thomas J. Hardy, Gareth J. Harrison, Ahmed Hassanin, Andrew T. Hattam, Martin Hein, Hytham K.S. Hmaid, Kay Hon, Kaisor Iqbal, Hakkı Z. Iscan, Arda Isik, Doireann P. Joyce, Maciej Juszczak, Kiriaki Kakavia, Stavros Kakkos, Christos D. Karkos, Emmanuel Katsogridakis, Rana Khalil, Andrew I. Khallaf, Aazeb Khan, Manar Khashram, Samantha Khoo, Joseph Kilby, Beatrice Kuang, Ioanna Kyrou, Pierfrancesco Lapolla, Kai W. Leong, Eunice Lim, Ju-wei N. Liu, Dafydd Locker, Xun Luo, Oliver T.A. Lyons, Ragai R. Makar, Dimitris Maras, Emmeline A. Martin, Hayrettin L. Mavioglu, Dennis Mazingi, James McCaslin, David N. McClure, Kevin McKevitt, Lewis Meecham, Shreya Mehta, Fabrizio Minelli, Andrea Mingoli, Afroditi M. Mitka, Farag S. Mohamed, Hayley M. Moore, Rachael L. Morley, Jana-Lee Moss, Konstantinos G. Moulakakis, Eustratia Mpaili, Ahmed Msherghi, Kamel Muhammad, Juanita Muller, Korana Musicki, Luis C.U. Nakano, Craig Nesbitt, Jonathan Nicholls, Andrew Nickinson, Thamer Nouh, Jose M.S. Nunag, Isaac K. Nyamekye, Michael J. Papanikolas, Theofanis T. Papas, Konstantinos O. Papazoglou, Sharath Paravastu, Noala Parr, Ketino Pasenidou, Fernando Picazo Pineda, Franklin Pond, Matthew A. Popplewell, Katarzyna Powezka, Daniela Prce, Sivaram Premnath, Raffaele Pulli, Hussein M.M. Rabee, Habibur P. Rahman, Nandhini Ravintharan, Andrés Reyes Valdivia, Toby Richards, Konstantinos Roditis, Alexander E.S. Rolls, Iain N. Roy, Hani Saeed, Prakash Saha, Alberto Saltiel, Paolo Sapienza, Emma Scott, Christopher N. Selvaraj, Atif Sharif, Simona Sica, Justinas Silickas, Gurkirat Singh, Ashwin Sivaharan, Yogeesan Sivakumaran, Pranav Somaiya, Gerry Stansby, Bethany M. Stavert, Abhilash Sudarsanam, Elizabeth Suthers, Helen Suttenwood, Ahmed Taha, Mohamed A.H. Taha, Siu C. Tam, Alethea M. Tang, Robert Tang, Dana Taran, Lawrence Tarusan, Myat S. Thet, Jacqueline Thomas, Sean Tierney, Konstantinos Tigkiropoulos, Giovanni Tinelli, Mahmoud M.H. Tolba, Hannah C. Travers, Ioannis Tsagkos, Yamume Tshomba, Paraskevi Tsiantoula, Christopher P. Twine, Berkay Ulker, Serap Ulusoy, Ertekin U. Unal, Vincent C. Varley, Thodur M. Vasudevan, Uyen G. Vo, Timothy Wagner, Stewart R. Walsh, Judy Wang, Jackie Wong, Sarah A. Warren, Chun L.P. Yih, Sergio Zacà, Adeel S. Zafar, Shady Zaki, Ewa M. Zywicka

**Affiliations:** a Nuffield Department of Population Health, University of Oxford, Oxford, UK; b Department of Vascular, Endovascular & Transplant Surgery, University of Otago, Christchurch, New Zealand; c South East Wales Vascular Network, UK; d Population Health Sciences Institute, Newcastle University, Newcastle Upon Tyne, UK; e University of Wolverhampton, Wolverhampton, UK; f University of Bristol, Bristol, UK; g Department of Vascular Surgery, Royal Victoria Hospital, Belfast, UK; h Department of Vascular Surgery, University Hospitals of Leicester NHS Trust, Leicester, UK; i Hull York Medical School, Hull, UK; j Imperial Vascular Unit, Imperial College Healthcare NHS Trust, London, UK; k Department of Surgery & Cancer, Imperial College London, London, UK; l NIHR Leicester Biomedical Research Centre, Leicester, UK; m South Tyneside and Sunderland Foundation Trust, Sunderland, UK; n Cambridge University Hospitals, Cambridge, UK

**Keywords:** Vascular surgery, COVID-19, Coronavirus, Mortality

## Abstract

**Objective:**

The first COVID-19 pandemic wave was a period of reduced surgical activity and redistribution of resources to only those with late stage or critical presentations. This Vascular and Endovascular Research Network COVID-19 Vascular Service (COVER) study aimed to describe the six-month outcomes of patients who underwent open surgery and or endovascular interventions for major vascular conditions during this period.

**Methods:**

In this international, multicentre, prospective, observational study, centres recruited consecutive patients undergoing vascular procedures over a 12-week period. The study opened in March 2020 and closed to recruitment in August 2020. Patient demographics, procedure details, and post-operative outcomes were collected on a secure online database. The reported outcomes at 30 days and six months were post-operative complications, re-interventions, and all cause in-hospital mortality rate. Multivariable logistic regression was used to assess factors associated with six-month mortality rate.

**Results:**

Data were collected on 3 150 vascular procedures, including 1 380 lower limb revascularisations, 609 amputations, 403 aortic, 289 carotid, and 469 other vascular interventions. The median age was 68 years (interquartile range 59, 76), 73.5% were men, and 1.7% had confirmed COVID-19 disease. The cumulative all cause in-hospital, 30-day, and six-month mortality rates were 9.1%, 10.4%, and 12.8%, respectively. The six-month mortality rate was 32.1% (95% CI 24.2–40.8%) in patients with confirmed COVID-19 compared with 12.0% (95% CI 10.8–13.2%) in those without. After adjustment, confirmed COVID-19 was associated with a three times higher odds of six-month death (adjusted OR 3.25, 95% CI 2.18–4.83). Increasing ASA grade (3–5 *vs*. 1–2), frailty scores 4–9, diabetes mellitus, and urgent and or immediate procedures were also independently associated with increased odds of death by six months, while statin use had a protective effect.

**Conclusion:**

During the first wave of the pandemic, the six-month mortality rate after vascular and endovascular procedures was higher compared with historic pre-pandemic studies and associated with COVID-19 disease.

## Highlights

• Six-month mortality after vascular procedures during the COVID-19 pandemic was 12.8%.
• Cumulative mortality rate was higher compared with pre-pandemic figures in the literature.
• Of the included cases, 3.7% had confirmed or suspected COVID-19.
• Confirmed COVID-19 was associated with a three times higher odds of six-month mortality.

## Introduction

Following the first Coronavirus Disease-19 (COVID-19) pandemic wave beginning in March 2020, the vascular community, along with colleagues in all specialties, experienced the effects of rising COVID-19 cases and hospital admissions, leading to a scarcity of resources and rationing of treatment. During the first wave of COVID-19, finite availability of healthcare services led to the release of international guidance for vascular surgeons. This guidance limited the type of vascular interventions recommended for each of the key vascular conditions. Surgery was recommended for only the most severe or late stage presentations of vascular disease (i.e., crescendo transient ischaemic attack; ischaemic limb with tissue loss or rest pain; and abdominal aortic aneurysm (AAA) > 6.5–7 cm [compared with the usual treatment threshold of 5.5 cm]).^1^^,^^2^

The Vascular and Endovascular Research Network (VERN) COvid-19 Vascular sERvice (COVER) study previously published the in-hospital outcome data for an international cohort of 1 103 patients who underwent surgical or endovascular intervention during the first pandemic wave in 2020.^3^ Results demonstrated that, despite documenting 4.0% suspected or confirmed COVID-19 amongst patients, in-hospital mortality following intervention for eligible aortic disease, lower limb revascularisation, major amputation, and carotid interventions exceeded pre-pandemic outcomes by tenfold. It was suggested that the increased mortality rate was due to delays in treatment, healthcare staff shortages, and reduced access to higher level care beds. By the end of the study period, complete records for 3 150 patients were available for analysis, including follow up data to six months. This paper aimed to report the six-month follow-up outcomes for these patients.

## Methods

### Study design

The VERN COVER study was a prospective, observational cohort study (ISRCTN registration reference number: 80453162) that aimed to capture procedural, post-operative, and follow-up information on vascular and endovascular interventions undertaken during the COVID-19 pandemic.^4^ The study was performed and is reported in accordance with the STROBE guidelines.^5^ United Kingdom (UK) National Health Service (NHS) Research Ethics Committee and Health Research Authority approval was granted prior to commencing recruitment (20/NW/0196 Liverpool Central) in March 2020. Centres outside the UK obtained institutional review board approval before participation, as per local and national regulations. Study sponsorship was provided by the research and development department, University Hospitals Coventry and Warwickshire, Coventry, UK. The study was conducted in line with the Declaration of Helsinki. Consent procedures were performed as per the guidance at each institution. De-identified data were transferred to a UK NHS server (based at the University of Birmingham) as per Health Research Authority and NHS principles. Data sharing agreements were used for all participating institutions. Each centre was required to record local identifiers on a secure, local, General Data Protection Regulation (GDPR) compliant database to allow longitudinal data capture and linkage, which was overseen by the study sponsor.

### Study cohort

Each recruiting site's investigators prospectively collected information on consecutive patients with a vascular pathology undergoing any vascular or endovascular procedure over a period of 12 weeks from local study opening, using an online purpose built database on Research Electronic Data Capture (REDCap).^6^ All patients who were entered into the database from the study opening on 20 March 2020 to 31 August 2020 were included in this study. Records set as incomplete by the local team entering data were excluded from the analysis.

### Patient characteristics

Baseline data that were prospectively recorded included: demographics, type of procedure, co-existing health conditions (including suspected or confirmed COVID-19 disease), medications being taken prior to the procedure (on admission or started acutely), American Society of Anesthesiologists (ASA) physical status classification, and frailty expressed by the Clinical Frailty scale (CFS).^7^ The CFS is a validated judgement-based tool that measures the functional status of individuals, including their mobility, use of walking aids, and ability to perform activities of daily living, with scores ranging from 1 (very fit) to 9 (terminally ill).^7^ Peri-procedural data that were collected included the time from presentation to intervention, mode of anaesthesia, type of operation, and post-operative care environment. Post-procedural data included unplanned admission to critical care, in-hospital death, total length of hospital stay, SARS-CoV-2 pneumonia (using each centre's own practice standards for diagnosis), and post-operative complications.

### Study outcomes

The primary outcome was all cause cumulative mortality at six months after the procedure. Secondary outcomes included six-month re-admission, re-intervention, myocardial infarction, respiratory complications, SARS-CoV-2 pneumonia, surgical site infection, and major amputation rates. In-hospital and 30-day cumulative outcomes were also reported.

### Statistical analysis

Normally distributed data are presented as mean and standard deviation, and non-normally distributed data as median and interquartile range (IQR). Multivariable logistic regression was used to assess risk factors for six-month death. The risk factors found to be statistically significant at the 90% confidence level in the univariable binary logistic regression analysis were taken forward to multivariable logistic regression; these included comorbidities (including COVID status), medications, ASA grade, frailty score, and urgency of surgery. The associations between the determinants and the outcomes in the logistic regression models were expressed as odds ratios with 95% confidence intervals. Statistical analysis was performed using STATA version 17 (StataCorp, College Station, TX, USA).

## Results

Of the 3 745 procedure records entered in the database, 267 had missing covariables (age, sex, or type of procedure) and 328 were marked as incomplete and excluded. Therefore, 3 150 records from 69 vascular centres in 21 countries were included in the analysis. Most of the submitted procedures were performed in the UK (*n* = 1 152, 36.6%) and Australia (*n* = 754, 23.9%) (Supplementary Table S1). Table 1 contains the baseline characteristics of the cohort overall and by type of procedure. The median age was 68 years (IQR 59, 76) and 2 315 patients (73.5%) were men. Fifty two patients (1.7%) were reported to have confirmed COVID-19 disease and 67 (2.1%) had suspected COVID-19 at the time of the procedure.

**Table 1:** Patient characteristics by procedure type.

Characteristic	Total (*n* =3 150)	Aortic (*n* = 403)	Carotid (*n* = 289)	Lower limb revascularisation (*n* = 1 380)	Amputation (*n* = 609)	Vascular Access (*n* = 258)
Age – years	68 (59, 76)	74 (68, 80)	72 (65, 78)	69 (61, 77)	65 (57, 73)	60 (48, 70)
Male sex	2 315 (73.5)	345 (85.6)	210 (72.7)	1 010 (73.2)	468 (76.8)	150 (58.1)
*Comorbidities*						
Diabetes – type 1 or 2	1 485 (47.1)	80 (19.9)	86 (29.8)	649 (47)	491 (80.6)	130 (50.4)
Hypertension	2 019 (67.0)	284 (70.5)	232 (80.3)	921 (66.7)	382 (62.7)	186 (72.1)
COPD	459 (14.6)	78 (19.4)	39 (13.5)	226 (16.4)	84 (13.8)	10 (3.9)
Myocardial infarction	702 (22.3)	112 (27.8)	60 (20.8)	329 (23.8)	141 (23.2)	35 (13.6)
Chronic kidney disease	645 (20.5)	58 (14.4)	21 (7.3)	218 (15.8)	146 (24)	168 (65.1)
Renal replacement/dialysis	299 (9.5)	8 (2)	1 (0.3)	59 (4.3)	45 (7.4)	176 (68.2)
Stroke/TIA	413 (13.1)	37 (9.2)	130 (45)	140 (10.1)	75 (12.3)	15 (5.8)
Cancer	192 (6.1)	40 (9.9)	15 (5.2)	84 (6.1)	33 (5.4)	7 (2.7)
Dementia	72 (2.3)	12 (3)	12 (4.2)	24 (1.7)	19 (3.1)	3 (1.2)
Peripheral artery disease	1 205 (38.3)	55 (13.6)	40 (13.8)	788 (57.1)	261 (42.9)	26 (10.1)
Current smoker	562 (17.8)	71 (17.6)	58 (20.1)	298 (21.6)	98 (16.1)	11 (4.3)
*ASA grade (8 missing)*						
1–2	572 (18.2)	74 (18.5)	72 (24.9)	242 (17.6)	83 (13.7)	31 (12.0)
3–5	2 570 (81.8)	327 (81.5)	217 (75.1)	1 135 (82.4)	524 (86.3)	227 (88.0)
*Frailty (84 missing)*						
Not frail (score 1–3)	1 527 (49.8)	271 (68.6)	197 (69.1)	605 (45.2)	201 (34.1)	124 (49.2)
Moderate (score 4–6)	1 399 (45.6)	118 (29.9)	85 (29.8)	684 (51.1)	327 (55.5)	123 (48.8)
Severe (score 7–9)	140 (4.6)	6 (1.5)	3 (1.1)	50 (3.7)	61 (10.4)	5 (2.0)
*COVID-19 disease*						
Confirmed	52 (1.7)	1 (0.2)	4 (1.4)	16 (1.2)	17 (2.8)	0 (0.0)
Suspected	67 (2.1)	5 (1.2)	8 (2.8)	13 (0.9)	18 (3.0)	7 (2.7)
*Urgency of surgery (18 missing)*						
Elective	981 (31.3)	197 (49.4)	105 (37.4)	395 (28.6)	65 (10.8)	160 (62.0)
Expedited	1 342 (42.9)	105 (26.3)	143 (50.9)	656 (47.6)	325 (53.8)	60 (23.3)
Urgent	646 (20.6)	52 (13)	31 (11)	259 (18.8)	191 (31.6)	37 (14.3)
Immediate	163 (5.2)	45 (11.3)	2 (0.7)	69 (5.0)	23 (3.8)	1 (0.4)

Data are presented as *n* (%) or median (IQR).

Abbreviations: COPD, chronic obstructive pulmonary disease; TIA, transient ischaemic attack; ASA, American Society of Anesthesiologists.

### Procedure characteristics

The most common procedures performed in the study period were lower limb revascularisations, followed by amputations and aortic procedures, vascular access interventions, and carotid procedures (Table 1). The remaining 211 records (6.7%) pertained to thoracic outlet, mesenteric, and venous procedures.

The most common indication for aortic surgery was AAA reaching size threshold (*n* = 213, 52.9%), followed by symptomatic AAA (*n* = 71, 17.6%), ruptured AAA (*n* = 4 6, 11.4%), rapid growing AAA (*n* = 40, 9.9%), and acute aortic syndrome or transection (*n* = 33, 8.2%). The mean diameter of AAA operated on during this period was 60.0 (SD 18.9) mm. Primary endovascular repair was performed in 68.2% of aortic interventions, and open surgery in 24.0% (*n* = 96). Revision procedures accounted for 31 cases (7.8%). Lower limb revascularisations consisted of 698 endovascular, 526 open surgical, and 156 hybrid procedures. The most common indication was chronic limb-threatening ischaemia (*n* = 735, 53.5%), followed by claudication (*n* = 279, 20.3%), acute limb ischaemia (*n* = 194, 14.1%), graft stenosis (*n* = 51, 3.7%), aneurysm (*n* = 49, 3.6%), uncontrolled infection (*n* = 35, 2.5%), and trauma (*n* = 31, 2.3%); there were 268 major amputations (44%). Carotid interventions were performed for asymptomatic disease in 98 cases (33.9%), 92 transient ischaemic attacks (31.8%), 74 stroke (25.6%), and 25 ocular symptoms (8.7%). The median time from index neurological event to surgery was nine (IQR 3, 24) days.

Most of the procedures were expedited (*n* = 1 342, 42.9%) or elective (*n* = 981, 31.3%), with the remaining being urgent (*n* = 646, 20.6%) and immediate (*n* = 163, 5.2%). Procedures were performed under general anaesthetic (GA) in 1 761 cases (56.1%), under local anaesthetic in 904 (28.8%), and the rest were performed under spinal, epidural, or peripheral nerve block. Deviation from the normal anaesthetic type was reported in 39 cases (1.2%), usually because an alternative was given instead of GA (*n* = 18) or GA was used instead of local anaesthetic (*n* = 10). Regarding the destination after surgery, 696 patients (22.1%) were admitted to level 2 or level 3 care (high dependency unit or intensive care unit, respectively), 2 091 returned to the ward (level 1 care) (66.5%), whereas 204 (6.5%) were treated as day cases. Deviation from the usual post-operative care environment was reported in 76 patients (2.4%), and involved patients treated in level 3 care instead of level 2 (*n* = 29) or the ward (*n* = 10), and others returning to the ward instead of level 2 (*n* = 10) or level 3 care (*n* = 5).

### In-hospital outcomes

During their hospital stay, 20.2% of patients (95% CI 18.8–21.6%) experienced a post-operative complication and 10.0% (95% CI 8.9–11.1%) returned to theatre for a further procedure. In-hospital mortality across all procedure types was 9.1% (95% CI 8.2–10.2%). In-hospital mortality for aortic procedures was highest at 11.4% (95% CI 8.5–14.9%), closely followed by amputations (11.0%, 95% CI 8.6–13.8%), carotid procedures (6.9%, 95% CI 4.3–10.5%), and lower limb revascularisation (7.3%, 95% CI 5.9–8.7%). The post-operative complications reported for each procedure type are detailed in Table 2. The median length of stay was the longest for amputations (median 10; IQR 5, 18 days) and shortest for vascular access procedures (median 2; IQR 1, 4 days).

**Table 2:** In-hospital events by type of procedure.

Event	Aortic	Carotid	Lower limb revascularisation	Amputation	Vascular access
Length of stay – days	5 (3, 10)	4 (2, 6)	6 (3, 12)	10 (5, 18)	2 (1, 4)
*Complications*					
Cardiac	28 (6.9)	2 (0.7)	48 (3.5)	29 (4.8)	3 (1.2)
Respiratory	42 (10.4)	5 (1.7)	51 (3.7)	32 (5.3)	1 (0.4)
Stroke	3 (0.7)	11 (3.8)	10 (0.7)	4 (0.7)	0 (0)
Renal failure	21 (5.2)	0 (0)	34 (2.5)	18 (3.0)	0 (0)
Bleeding	18 (4.5)	12 (4.2)	60 (4.4)	13 (2.1)	7 (2.7)
Surgical site infection	3 (0.7)	2 (0.7)	82 (5.9)	69 (11.3)	1 (0.4)
Limb ischaemia	10 (2.5)	0 (0)	103 (7.5)	17 (2.8)	1 (0.4)
Any complication	102 (25.3)	29 (10.0)	306 (22.2)	151 (24.8)	12 (4.7)
Return to theatre	29 (7.2)	8 (2.8)	180 (13.0)	70 (11.5)	12 (4.7)
Death	46 (11.4)	20 (6.9)	100 (7.3)	67 (11.0)	26 (10.1)

Data are presented as *n* (%) or median (interquartile range).

### 30-day outcomes

The 30-day outcomes for patients undergoing vascular procedures during the first COVID-19 pandemic wave are shown in Table 3. Fifty eight patients underwent a major amputation within 30 days of a revascularisation procedure (4.2%, 95% CI 3.2–5.4%). A total of 1.8% of patients had SARS-CoV-2 pneumonia (95% CI 1.4–2.3%), with the rate of pneumonia due to other pathogens being 2.4% (95% CI 1.9–3.0%). Pneumonia was most frequent after aortic procedures (5.7%, 95% CI 3.7–8.4%). The cumulative all cause mortality rate at 30 days was 10.4% (95% CI 9.3–11.5%), highest for patients with primary amputation (12.3%, 95% CI 9.8–15.2%), and lowest for patients undergoing carotid interventions (8.0%, 95% CI 5.1–11.7%) (Table 3). The 30-day re-admission rate was 11.0% overall (95% CI 9.9–12.2%), and was highest following vascular access procedures (15.1%, 95% CI 11.0–20.1%) and lower limb revascularisation (12.3%, 95% CI 10.6–14.1%).

**Table 3:** Thirty-day outcomes by type of procedure.

Outcome	Aortic	Carotid	Lower limb revascularisation	Amputation	Vascular access
Myocardial infarction	12 (3.0)	3 (1.04)	27 (2.0)	9 (1.5)	2 (0.8)
SARS-CoV-2 pneumonia	3 (0.7)	5 (1.7)	24 (1.7)	13 (2.1)	2 (0.8)
Pneumonia of other cause	23 (5.7)	4 (1.4)	26 (1.9)	21 (3.5)	2 (0.8)
SSI not requiring admission	4 (1.0)	2 (0.7)	42 (3.0)	27 (4.4)	1 (0.4)
SSI requiring admission	5 (1.2)	1 (0.4)	71 (5.1)	41 (6.7)	6 (2.3)
Graft or stent occlusion	7 (1.7)	4 (1.4)	42 (3.0)	-	6 (2.3)
Major amputation	0 (0)	-	58 (4.2)	-	-
Re-admission	36 (8.9)	16 (5.5)	169 (12.3)	68 (11.2)	39 (15.1)
Mortality	48 (11.9)	23 (8.0)	119 (8.6)	75 (12.3)	29 (11.2)

Data are presented as *n* (%).

Abbreviation: SSI = surgical site infection.

### Cumulative six-month outcomes

Six-month outcomes by procedure type are documented in Table 4. Any type of re-intervention was required in 18.2% of patients (95% CI 16.8–19.6%) and re-admission occurred in 22.5% (95% CI 21.1–24.0%). The most common complication was surgical site infection (15.3%, 95% CI 14.1–16.6%), most of which were diagnosed after discharge (10.1%, 95% CI 9.1–11.2%). Other complications included non-SARS-CoV-2 pneumonia documented after 5.9% of procedures (95% CI 5.1–6.8%), graft or stent occlusion in 4.4% (95% CI 3.7–5.1%), myocardial infarction in 4.9% (95% CI 4.2–5.7%), and stroke in 1.4% (95% CI 1.0–1.9%). At six months after a lower limb revascularisation procedure, 7.8% of patients (95% CI 6.4–9.3%) had gone on to have a major amputation.

**Table 4:** Six-month outcomes by type of procedure.

Outcome	Aortic	Carotid	Lower limb revascularisation	Amputation	Vascular access
Myocardial infarction	31 (7.7)	4 (1.4)	68 (4.9)	37 (6.1)	4 (1.6)
Stroke	5 (1.2)	11 (3.8)	19 (1.4)	7 (1.2)	0 (0.0)
SARS-CoV-2 infection	16 (4.0)	16 (5.5)	64 (4.6)	46 (7.6)	14 (5.4)
SARS-CoV-2 pneumonia	6 (1.5)	7 (2.4)	33 (2.4)	13 (2.1)	2 (0.8)
Pneumonia of other cause	48 (11.9)	7 (2.4)	69 (5.0)	52 (8.5)	2 (0.8)
SSI not requiring admission	8 (2.0)	2 (0.7)	80 (5.8)	47 (7.7)	1 (0.4)
SSI requiring admission	10 (2.5)	1 (0.4)	138 (10.0)	93 (15.3)	6 (2.3)
Graft or stent occlusion	14 (3.5)	4 (1.4)	94 (6.8)	1 (0.2)	17 (6.6)
Major amputation	-	-	107 (7.8)	-	-
Re-intervention	43 (10.7)	8 (2.8)	325 (23.6)	125 (20.5)	46 (17.8)
Re-admission	68 (16.9)	18 (6.2)	350 (25.4)	170 (27.9)	72 (27.9)
Death	55 (13.6)	24 (8.3)	159 (11.5)	95 (15.6)	36 (14.0)

Data are presented as *n* (%).

Abbreviation: SSI = surgical site infection.

The all cause cumulative mortality rate at six months was 12.8% (95% CI 11.6–14.0%) overall; 32.1% (95% CI 24.2–40.8%) in patients with confirmed COVID-19 compared with 12.0% (95% CI 10.8–13.2%) in those without. Aortic procedures had an overall documented mortality rate of 13.6% (95% CI 10.4–17.4%), three times higher in urgent (28.8%, 95% CI 17.1–43.1%) or emergency cases (35.6%, 95% CI 21.9–51.2%) compared with elective or scheduled procedures (7.9%, 95% CI 5.2–11.6%). Outcomes by indication and urgency of surgery for AAA procedures are presented in Supplementary Table S2. Patients with uncontrolled infection had a mortality rate of 31.4% (95% CI 16.9–49.3%), the highest among all indications for revascularisation, followed by acute limb ischaemia (17.0%, 95% CI 12.0–23.1%), chronic limb-threatening ischaemia (10.3%, 95% CI 8.2–12.8%), and claudication (8.6%, 95% CI 5.6–12.5%). The six-month mortality rate following major amputation was found to be 17.9% (95% CI 13.5–23.0%) and 13.8% (95% CI 10.3–17.9%) following minor amputations. Procedures for asymptomatic carotid disease were associated with a 10.2% (95% CI 5.0–18.0%) six-month mortality rate, while the rate was 7.3% (95% CI 4.1–12.0%) for symptomatic carotid disease. Six-month mortality figures by procedure type for the eight countries that submitted >100 procedures in total are presented in Supplementary Table S3.

**Figure 1:**
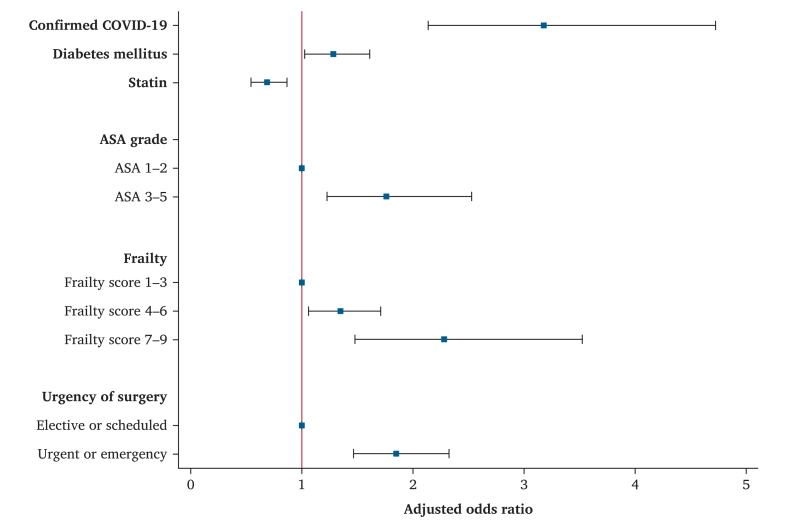
Adjusted model of predictors for six-month mortality rate, including data from the 3 044 patients with completed six-month recorded outcomes. ASA: American Society of Anaesthesiologists.

After adjusting for ASA grade, frailty, diabetes status, urgency of procedure, and statin administration, confirmed COVID-19 disease was associated with three times higher odds of six-month death (adjusted OR 3.25, 95% CI 2.18–4.83) (Supplementary Table S4). In the multivariable model, increasing ASA grade (3–5 *vs*. 1–2), moderate and severe frailty, presence of diabetes mellitus, and urgent or immediate procedure were also independently associated with increased odds of death at six months, while statin use had the opposite effect (Fig. 1).

### One-year follow up

One year follow up data were available for 2 396 patients (76.1%). All cause cumulative one-year mortality was 14.4% (95% CI 13.0–15.9%). One-year outcomes for this subgroup by procedure type are presented in Supplementary Table S5.

## Discussion

This six-month follow up analysis of the original COVER study has shown that, amongst 3 150 patients undergoing vascular procedures during the first wave of the COVID-19 pandemic, the six-month mortality rate was similar to the in-hospital mortality rate, suggesting that most deaths occurred at the time of the index procedure.

The all cause mortality rate at six months post-operatively was consistently higher than pre-pandemic levels reported in the literature for all procedural groups, despite there being a relatively low COVID-19 disease incidence in this cohort.^8^, ^9^, ^10^, ^11^, ^12^ It is unclear why this was the case, but is hypothesised that the pandemic could have had secondary consequences such as delays in procedures, late presentation of patients, operating only on more severe or urgent cases, and disruptions to vascular service delivery and established pathways. It is also possible that the true incidence of infection was higher than identified in this study due to varying testing policies and the lack of availability of COVID-19 tests during the first stages of the pandemic; it is estimated that approximately 10% of the UK's population had SARS-CoV-2 antibodies in April 2020.^13^

Despite low rates of documented infection, the results from this study support the previously reported high mortality rate observed in patients undergoing vascular procedures following a positive COVID-19 test.^14^^,^^15^ The multivariable regression analysis demonstrated that peri-operative SARS-CoV-2 infection was associated with an increased risk of death at six months by a factor of 3.3, after adjusting for other independent predictors of six-month mortality, such as increasing ASA,^16^ increased frailty,^17^, ^18^, ^19^ diabetes mellitus,^20^ and urgent surgery.^21^

The in-hospital mortality rate for this cohort of 3 150 patients was lower than that observed for the 1 103 patients included in the previous report overall (9.1% *vs*. 11.0%) and for each procedure separately, with the greatest difference observed in aortic (11.4 *vs*. 15.2% previously) and carotid procedures (6.9 *vs*. 10.7% previously).^3^ The previous publication included 1 103 patients who were recruited earlier in the COVID-19 pandemic period, suggesting that the additional mortality risk may have decreased with time, possibly due to the services adapting as the pandemic progressed. The two cohorts were comparable in terms of patients with suspected or confirmed SARS-CoV-2 infection (3.7 *vs*. 4.0%) and the proportion of elective or expedited procedures was slightly increased in the larger cohort (73.7 *vs*. 71.6%).

Like aortic procedures, death following carotid procedures seemed to occur early in the post-operative period in both symptomatic and asymptomatic patients, and was higher than pre-pandemic levels;^22^, ^23^, ^24^ the reason for this is unclear and the study did not collect information on the cause of death. It was hypothesised that the known differences in stroke and death rates following carotid procedures in both randomised and real world data, and the importance of tight and rapid blood pressure control^25^ that is more reliably achieved in level 2 and 3 care, could potentially explain the excess strokes and deaths observed in this cohort with a relatively low incidence of COVID-19. The high observed mortality rate could also be partly due to the sequelae of undiagnosed SARS-CoV-2 infection, if the true incidence was higher in the early pandemic period that this study covered.

Conversely, patients undergoing lower limb revascularisation or amputation procedures had a six-month mortality rate equivalent to that reported in the literature in the years preceding the pandemic.^26^^,^^27^ This could also reflect the relatively high mortality risk associated with the disease process rather than the procedure itself in these patients.^28^ It was noted that despite international guidance regarding the deferral of non-urgent procedures such as revascularisations for claudication, a small proportion of these were still performed during the first wave of the pandemic. This could be explained by country specific and other local guidance, which was usually informed by the spread of the virus in each country and the lockdown or other containment measures that were put in place.

The COVID-19 pandemic disrupted operating theatre activity across all surgical specialties, and increased morbidity and mortality has been reported by other studies. A systematic review and meta-analysis of studies on neurosurgical procedures reported significantly increased mortality during the pandemic.^29^ Studies on general surgical procedures have also found higher post-operative complications in SARS-CoV-2 infected patients, higher failure to rescue in non-infected patients,^30^ and higher in-hospital mortality in patients operated on during that period irrespective of infection status.^31^

This study had some limitations. First, the observational nature meant that causality could not be inferred, and there was probably residual confounding. A difficulty of undertaking the study during the pandemic was that practices (including testing provision and policy) probably considerably varied within and between countries, and the proportion of patients tested for SARS-CoV-2 infection is unknown. Data were reported by the clinicians in each participating centre, potentially limiting the accuracy, and there was a risk of data entry errors. Similarly, due to the need to consent patients for inclusion, it cannot be guaranteed that patients were enrolled in a consecutive manner. This may have also led to an under representation of mortality if there was insufficient time or resources to consent patients in the emergency setting. Finally, there were no available historical data of the pre-COVID-19 pandemic period from each centre; therefore, it was not possible to make direct comparisons and draw robust conclusions regarding the difference in outcomes over time.

### Conclusion

In-hospital death during the first wave of the COVID-19 pandemic was higher than pre-pandemic levels reported in the literature for most vascular surgery procedures, an effect which persisted up to six months. There is limited evidence on the causes of the reported mortality rate, but possible causes are the secondary consequences of the pandemic, including delays in treatment and competition for life preserving resources such as level 2 and 3 care.

## Funding

This work was supported by the Circulation Foundation. The National Institute for Health and Care Research (NIHR) provided salary support for the co-chief investigators (reference: NIHR000359) and study leads.

## Conflicts of interest

The authors have no conflicts of interest to declare. The funders of the study had no role in study design, data collection, data analysis, data interpretation, or writing of the report. The corresponding author and analysis group had full access to all the data in the study, and the corresponding author and the writing committee had final responsibility for the decision to submit for publication.
