# Supplementary material for: Outcomes Following Vascular and Endovascular Procedures Performed During the First COVID-19 Pandemic Wave
Source: EJVES Vasc Forum. 2024 Sep 19;62:64–71. doi: 10.1016/j.ejvsvf.2024.08.002 (PMC11462031; doi:10.1016/j.ejvsvf.2024.08.002)
Supplement: Multimedia component 1 [file mmc1.pdf]

Supplementary Table S1. Country of recruitment of study participants.

Country	Number of procedures	%
Great Britain	1152	36.57
Australia	754	23.94
Italy	268	8.51
Greece	219	6.95
Hong Kong	148	4.70
Saudi Arabia	115	3.65
USA	114	3.62
Spain	101	3.21
Bahrain	50	1.59
Egypt	47	1.49
Austria	35	1.11
Malaysia	31	0.98
Lybia	30	0.95
Sudan	22	0.70
Israel	13	0.41
Sri Lanka	13	0.41
New Zealand	11	0.35
Turkey	10	0.32
Ireland	8	0.25
Brazil	5	0.16
Jordan	4	0.13
Total	3150	100
