# Supplementary material for: Outcomes Following Vascular and Endovascular Procedures Performed During the First COVID-19 Pandemic Wave
Source: EJVES Vasc Forum. 2024 Sep 19;62:64–71. doi: 10.1016/j.ejvsvf.2024.08.002 (PMC11462031; doi:10.1016/j.ejvsvf.2024.08.002)
Supplement: Multimedia component 2 [file mmc2.pdf]

Supplementary Table S2. Six month outcomes of aortic procedures performed for abdominal aortic aneurysms (*n* = 370) stratified by indication and urgency of the procedure.

	**Elective/ expedited**	**Urgent non-ruptured**	**Ruptured**
**30-day outcomes**			
Non-SARS-CoV-2 pneumonia	9 (3.3)	4 (9.1)	8 (17.4)
Mortality	18 (6.5)	10 (22.7)	16 (34.8)
**6-month outcomes**			
Mortality	21 (7.6)	12 (27.3)	17 (37.0)
Myocardial infarction	14 (5.1)	5 (11.4)	9 (19.6)
Non-SARS-CoV-2 pneumonia	23 (8.3)	4 (9.1)	15 (32.6)
Re-intervention	25 (9.0)	5 (11.4)	9 (19.6)

Data are presented as n (%).

Indications for surgery for elective/expedited and urgent non-ruptured aneurysms included reaching size threshold, rapid growth, or being symptomatic.
