# Supplementary material for: Outcomes Following Vascular and Endovascular Procedures Performed During the First COVID-19 Pandemic Wave
Source: EJVES Vasc Forum. 2024 Sep 19;62:64–71. doi: 10.1016/j.ejvsvf.2024.08.002 (PMC11462031; doi:10.1016/j.ejvsvf.2024.08.002)
Supplement: Multimedia component 3 [file mmc3.pdf]

Supplementary Table S3. Six month mortality (%) by procedure type and country for the eight countries that contributed over 100 procedures in total in this study.

	Aortic	Carotid	Lower limb revascularisation	Amputation	Vascular access
Great Britain	10.2	4.7	11.5	14.9	21.2
Australia	9.2	5.5	9.3	13.3	10.1
Italy	19.6	4.7	6.6	36.8	n/a
Greece	20.4	11.4	22.4	21.1	n/a
Hong Kong	2.8	n/a	2.8	n/a	3.5
Saudi Arabia	n/a	n/a	29.0	10.5	31.7
USA	n/a	n/a	7.4	10.3	n/a
Spain	11.8	n/a	7.8	0.0	7.7

Data are presented as percentages and are shown if 10 or more procedures of a specific type were submitted, otherwise the percentage is replaced by n/a. Frequencies are not shown to prevent de-identification due to small numbers.
