# Supplementary material for: Outcomes Following Vascular and Endovascular Procedures Performed During the First COVID-19 Pandemic Wave
Source: EJVES Vasc Forum. 2024 Sep 19;62:64–71. doi: 10.1016/j.ejvsvf.2024.08.002 (PMC11462031; doi:10.1016/j.ejvsvf.2024.08.002)
Supplement: Multimedia component 4 [file mmc4.pdf]

Supplementary Table S4. Odds ratio and 95% confidence intervals for six month mortality from a multivariable model.

	Odds Ratio	95% CI	p-values
**Confirmed COVID-19**	3.18	2.14-4.73	<.001
**Diabetes mellitus**	1.29	1.03-1.61	.029
**Statin**	0.69	0.55-0.87	.002
**ASA grade**			.002
ASA 1-2	1.00	-	
ASA 3-5	1.76	1.23-2.53	
**Frailty**			<.001
Frailty score 1-3	1.00	-	
Frailty score 4-6	1.35	1.06-1.71	
Frailty score 7-9	2.28	1.48-3.53	
**Urgency**			<.001
Elective or Scheduled	1.00	-	
Urgent or Emergency	1.85	1.47-2.33	
