# Supplementary material for: Outcomes Following Vascular and Endovascular Procedures Performed During the First COVID-19 Pandemic Wave
Source: EJVES Vasc Forum. 2024 Sep 19;62:64–71. doi: 10.1016/j.ejvsvf.2024.08.002 (PMC11462031; doi:10.1016/j.ejvsvf.2024.08.002)
Supplement: Multimedia component 5 [file mmc5.pdf]

Supplementary Table S5. One year outcomes of 2 396 patients with submitted one year follow up data.

	Aortic	Carotid	Lower limb revascularisation	Amputation	Vascular access
Myocardial infarction	27 (8.5)	2 (0.9)	43 (4.2)	32 (7.1)	2 (1.2)
Stroke	3 (1.0)	6 (2.6)	10 (1.0)	6 (1.3)	0 (0)
SARS-CoV-2 pneumonia	6 (1.9)	8 (3.4)	36 (3.5)	16 (3.6)	2 (1.2)
Pneumonia of other cause	37 (11.7)	4 (1.7)	56 (5.4)	34 (7.5)	1 (0.6)
SSI not requiring admission	8 (2.5)	2 (0.9)	75 (7.2)	46 (10.2)	0 (0)
SSI requiring admission	7 (2.2)	0 (0)	96 (9.3)	57 (12.6)	2 (1.2)
Graft/stent occlusion	10 (3.2)	3 (1.3)	70 (6.8)	-	6 (3.6)
Major amputation	-	-	87 (8.4)	-	-
Re-intervention	30 (9.5)	6 (2.6)	224 (21.6)	72 (16.0)	18 (10.7)
Readmission	46 (14.6)	13 (5.6)	231 (22.3)	105 (23.3)	25 (14.8)
Mortality	50 (15.8)	21 (9.0)	138 (13.3)	76 (16.9)	27 (16.0)

Data are presented as n (%).
